# Teachers’ emotional labor in parent–teacher interactions: key elements and theoretical logic from a grounded theory approach

**DOI:** 10.3389/fpsyg.2025.1639197

**Published:** 2025-08-05

**Authors:** Li Zhai, Chen Guo

**Affiliations:** School of Educational and Psychological Science, Hefei Normal University, Hefei, China

**Keywords:** teachers’ emotional labor, parent–teacher interactions, key elements, theoretical logic, grounded theory

## Abstract

In the context of home–school cooperation becoming an important part of global education reform, the burdens of teachers’ emotional labor in parent–teacher interactions has increased. Their emotional labor in parent–teacher interactions not only affects home–school cooperation but also influences teachers’ well-being both professionally and personally. In response to the challenge of growing emotional labor burdens on teachers, the research on the key elements and theoretical logic of teachers’ emotional labor in parent–teacher interactions is of great significance. Semi-structured interviews were held with 20 teachers in primary and secondary schools in China. Retrieved data were coded at three levels to explore the key elements of teachers’ emotional labor in parent–teacher interactions and construct a theoretical model of these elements. The model is structured around seven elements: emotional labor environment, personal characteristics, emotion competencies, emotional foundation, emotional interactions, emotional labor practices, and emotional labor consequences. The findings construct a theoretical framework for teachers’ emotional labor in parent–teacher interactions, enrich research on this topic from a relational dimension, and provide theoretical and practical insights.

## 1 Introduction

### 1.1 Background of the study

Parent–teacher interaction refers to the two-way communication process between parents and teachers, which aims to promote children’s development. It serves as the foundation for building successful home–school partnerships (Leenders et al., 2019; Epstein and Sanders, 2006). Parent–teacher interactions are regarded as emotional practices (Lasky, 2000). It is essential for teachers in their interactions with parents to conduct emotion management “to create a publicly observable facial and bodily display” (i.e., emotional labor) (Hochschild, 1983). In workplaces, teachers are required to present emotions that are appropriate for educational goals (Yin and Lee, 2012; Morris and Feldman, 1996; Alemdar and Anılan, 2022). Nowadays, home–school cooperation has become an important component of educational reforms in countries around the world. As frontline agents for implementing change in schools, teachers, when facing educational reform, increased student diversity and administrative pressures, have to deal with more and more complex parent–teacher interactions than ever before (Nath and Pandey, 2025; Addi-Raccah and Arviv-Elyashiv, 2008). This increases the burden of emotional labor on teachers in their interactions with parents (Kinman et al., 2011; Akın et al., 2014; Kariou et al., 2021) and may make teachers more vulnerable (Chen and Cheng, 2022). Teachers’ emotional labor not only affects teachers’ work performance and job satisfaction, but also is tied to teachers’ well-being both professionally and personally (Wang et al., 2024). This study, which aims to explore teachers’ emotional labor in parent–teacher interactions, will contribute to facilitating parent–teacher interactions, improving home–school cooperation, and enhancing teachers’ well-being, and is therefore of great significance.

### 1.2 Research gap

Emotional labor was first proposed by Hochschild (1983) to refer to how service-sector workers regulate their emotions to meet professional expectations. Scholars found that teaching is also a form of emotional labor, as teachers are required to manage their emotions during interactions with students, parents, colleagues, and others (Hargreaves, 1998; Winograd, 2003; Isenbarger and Zembylas, 2006). Teachers’ emotional labor is defined as their management of feelings and publicly-expressed emotions in response to institutional and professional guidelines (Yin and Lee, 2012). Since emotional labor in teaching is generally regarded as stemming mainly from teacher–student interactions (Yin et al., 2017), research on such labor in classroom is abundant (Sutton, 2004; Taxer and Frenzel, 2015; Yin et al., 2017; Wang et al., 2019; Buric and Frenzel, 2020). However, studies on teachers’ emotional labor in parent–teacher interactions are relatively rare (Lasky, 2000; Kang et al., 2023). These studies have focused on issues such as emotional experiences in parent–teacher interactions (Hargreaves and Lasky, 2004; Frenzel et al., 2016), emotional display rules in parent–teacher interactions (Zembylas, 2005; Winograd, 2003), the influencing factors and strategies of teachers’ emotional labor in parent–teacher interactions (Zembylas, 2002; Cross and Hong, 2012). The existing studies are rather fragmented and scattered, and they lack systematic exploration of the elements of teachers’ emotional labor in parent–teacher interactions. This study tackles this deficit by employing the grounded theory approach. This method is particularly suitable for exploring new fields or areas with limited research (Strauss and Corbin, 1997). Moreover, grounded theory is a particularly useful method for understanding micro-level, action-oriented social interaction processes (Strauss and Corbin, 1997). For this reason, this study adopts grounded theory to induce and generate theories from data, explores the key elements of such emotional labor and their logical relationships, so as to fill the existing research gap.

### 1.3 Research question

This study aims to provide a reference theoretical framework for a comprehensive and in-depth understanding of the process of teachers’ emotional labor in parent–teacher interactions. The research objective is to provide a systematic reference for improving teachers’ emotional labor in parent–teacher interactions, thereby facilitating home–school cooperation and enhancing teachers’ well-being.

To achieve this objective, the study addresses the following research questions.

1. What are the key elements of teachers’ emotional labor in parent-teacher interactions?
2. How do the key elements affect their emotional labor?
3. What is the theoretical logic among these key elements?

## 2 Literature review

### 2.1 Emotions experienced by teachers in parent–teacher interactions

The emotions experienced by teachers in parent–teacher interactions can range from negative emotions to positive emotions. The negative emotions include depression, anxiety, disgust, anger, anxiety, frustration (Hargreaves and Lasky, 2004; Sutton and Wheatley, 2003; Becker et al., 2015; Buric et al., 2018) and the positive emotions include happiness, pride and enjoyment (Sutton and Wheatley, 2003; Chen, 2016). The emergence of these emotions is mostly due to factors such as parents, educational reforms, or social environment. The respect, support, and understanding from parents evoke teachers’ positive emotions such as joy and happiness (Sutton and Wheatley, 2003; Chen, 2016). Irresponsibility, doubt, excessive expectations, and unreasonable accusations from parents can trigger teachers’ negative emotions such as anger, frustration, anxiety (Hargreaves and Lasky, 2004; Chen, 2016; Chen and Wang, 2011). Curriculum reform policies can affect teachers’ emotions. If parents do not support the implementation of new teaching methods and fail to understand teachers’ emotions, teachers may experience unpleasant emotions such as anxiety and confusion (Chen, 2016; Lee and Yin, 2011). In addition, social and public criticism can affect teachers’ emotional experiences in parent–teacher interactions (Chen, 2016).

### 2.2 Emotional display rules of teachers in parent–teacher interactions

The emotional display rules of teachers in parent–teacher interactions reflect the social requirements of what teachers should do and avoid when dealing with parents (Zembylas, 2002). In parent–teacher interactions, teachers should demonstrate positive emotions (such as enjoyment), conceal negative emotions (such as anxiety), and maintain the emotional intensity at a medium level (Cross and Hong, 2012; Becker et al., 2015; Sutton, 2004; Cowie, 2011). Teachers are expected to maintain emotional detachment and avoid incorporating personal emotions when they interact with parents (Lasky, 2000; Grumet, 1988; Wu and Song, 2023) in order to present the rational image of teachers as professionals. Teachers should interact with parents empathetically, showing initiative and kindness. When facing challenging parents, they should adopt an understanding and compassionate attitude (Leenders et al., 2019; Grumet, 1988; Liu, 2012).

### 2.3 Factors affecting teachers’ emotional labor in parent–teacher interactions

In previous studies, two approaches have been used to investigate the influencing factors of teachers’ emotional labor in parent–teacher interactions. One approach embodies Grandey’s conceptual framework (Grandey, 2000), suggesting that there are three factors: situational cues, organizational factors and personal factors. Situational cues not only include positive or negative emotional events (Goldberg and Grandey, 2007; Liang, 2019) but also include the ways in which teachers interact with parents (Farouk, 2012). Organizational factors such as school policy (Darby et al., 2011), school cultural environment (Cross and Hong, 2012), working conditions (Barutçu and Serinkan, 2013; Hassard et al., 2017) also impact their emotional labor. Personal factors such as teachers’ gender, personality, teaching age, qualifications, professional awareness, emotional intelligence affect teachers’ emotional labor in parent–teacher interactions (Basim et al., 2013; Yin et al., 2017; Gonzales, 2022). These studies reflect the perspectives on emotional labor from the work levels and the individual level (Brotheridge and Grandey, 2002). Another approach is to draw on insights from emotional geography (Hargreaves, 2001; Hargreaves and Lasky, 2004), that is, from the perspectives of sociocultural geography, moral geography, professional geography, political geography, and physical geography, which highlights the influence of factors such as parents’ sociocultural status, teachers’ moral purposes, teachers’ perceptions of boundaries between schools and homes, hierarchical power structures in parent–teacher interactions, and the frequency of parent–teacher contact (Chen and Wang, 2011; Dotger et al., 2011; Zembylas, 2015; Stroetinga et al., 2019).

### 2.4 Teachers’ emotional labor strategies in parent–teacher interactions

Teachers’ emotional labor strategies in parent–teacher interactions can be divided into surface acting, deep acting, and expression of naturally felt emotions, which conforms to the general classification of teachers’ emotional labor strategies (Hochschild, 1983; Yin, 2012). Surface acting refers to the strategy in which teachers fake unfelt emotions or hide felt emotions in order to display the appropriate emotions in school. In contrast, deep acting means the process in which teachers try to modify their felt emotions by employing certain cognitive techniques to achieve the desired emotional displays. Expression of naturally felt emotions reflects the possibility that teachers spontaneously experience and display the appropriate emotions required by the work (Yin, 2012). Some scholars, through qualitative case studies, have found that teachers frequently employ surface acting during interactions with parents (Rayner and Espinoza, 2015). Additionally, quantitative research by other scholars has revealed that elementary school teachers engage in more surface acting and deep acting compared to traditional high school teachers. They also exhibit more natural emotions than both traditional and vocational high school teachers (Yılmaz et al., 2015). These studies indicate that teachers use different emotional labor strategies in teacher - parent interactions. Yet, scholars lack in - depth research on the reasons why these various strategies are employed.

In sum, significant achievements have been made in related researches, yet there are two deficiencies. One deficiency is the insufficient attention paid to the uniqueness of teachers’ emotional labor in parent–teacher interactions. Teachers, as knowledge-based service (KBS) workers, differ from service employees in that they enjoy considerable autonomy in their work and maintain relatively stable relationships with stakeholders such as students, parents, and colleagues (Yin et al., 2019). Moreover, unlike the interactions between teachers and students, which are built on a teaching-and-learning relationship, the interactions between teachers and parents are established based on a collaborative partnership where both parties work together for children’s development (Gao, 2022). This fundamental difference in the nature of the relationship means that teachers’ emotional labor in parent–teacher interactions carries its own distinctive features, which have not yet received sufficient attention (Lasky, 2000; Kang et al., 2023; Stroetinga et al., 2019). Only by examining teachers’ emotional labor in parent–teacher interactions on the basis of grasping the characteristics of such interactions, and by systematically exploring the key elements of teachers’ emotional labor as well as the logical connections between these elements, can a comprehensive and in-depth understanding of teachers’ emotional labor within these interactions be achieved. Another deficiency lies in the fact that the research methods need to be enriched. Although scholars have employed both qualitative and quantitative methods to explore teachers’ emotional labor in parent–teacher interactions, previous related studies remain fragmented and have failed to develop a systematic understanding of teachers’ emotional labor in such interactions or to establish a coherent theoretical framework. Grounded theory, by identifying core concepts and their relationships that reflect social processes or phenomena from empirical data, enables the construction of theories from the bottom up (Glaser and Strauss, 1967). Therefore, this study adopts grounded theory to summarize the key elements of teachers’ emotional labor in parent–teacher interactions, distill its theoretical logic, and construct a theoretical framework.

## 3 Method and data

### 3.1 Research method

Grounded theory method is a qualitative research method proposed by Barney G. Glaser and Anselm L. Strauss (Glaser and Strauss, 1967). Grounded theory conducts induction on data without preconceived notions for the purpose of discovering theory (Corbin and Strauss, 2008). This method, which employs specific techniques for data classification and feature identification, is also particularly suitable for researching interpersonal interaction processes (Glaser and Strauss, 1998). The objective of this study is to generalize key elements of teachers’ emotional labor in parent–teacher interactions based on data and construct a theoretical model of the elements, which makes grounded theory method an apt choice for this research.

**Figure 1:**
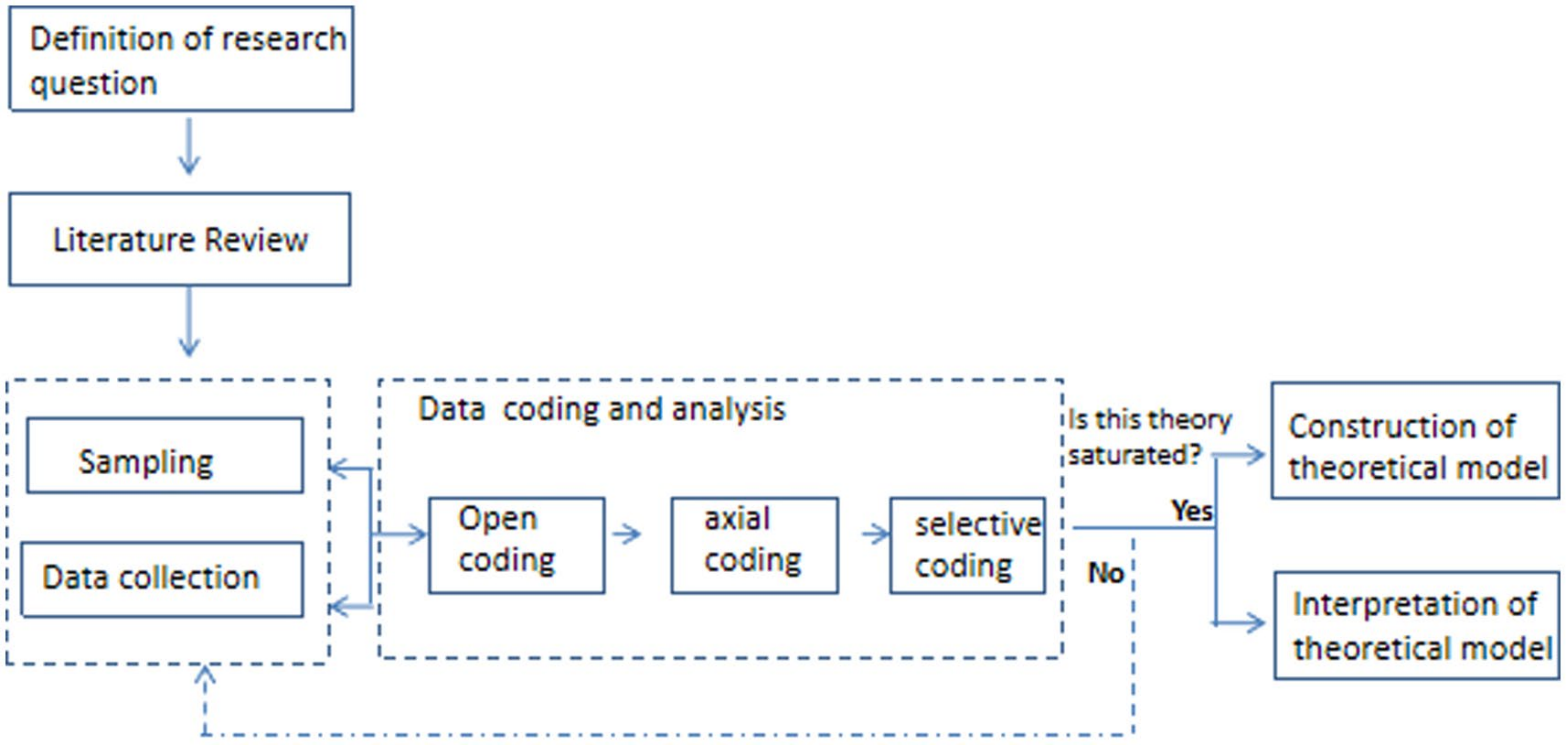
The flowchart based on grounded theory.

The flowchart of this study is shown in Figure 1. This study selects interview samples on the basis of posing questions and conducting a literature review, collects data by semi-structured interviews, then proceeds with open coding, axial coding, and selective coding of the interview texts in sequence. By systematically analyzing the data through these coding procedures, the study summarizes the key elements of teachers’ emotional labor in parent–teacher interactions, distills the theoretical logic among these elements. After a theoretical saturation test, the key elements and theoretical model of teachers’ emotional labor in parent–teacher interactions are constructed, followed by an interpretation of the theoretical model.

### 3.2 Sampling and data collection

The interviews were conducted from October 2023 to May 2024, and a total of 20 head teachers of primary and secondary schools in China were interviewed. In primary and secondary education, head teachers, due to their job responsibilities, tend to have more interactions with parents and also engage in more emotional labor. Therefore, head teachers were chosen as the interviewees as to obtain the maximum amount of information needed for this research. When selecting samples, this study also consider the diversity of samples and the convenience of researchers. Table 1 gives the characteristics of the samples, including region, school, gender, education, years of service as a head teacher, marital status and fertility status.

**Table 1:** Characteristics of the samples (*N* = 20).

Characteristic	No. of Participants	Percentage
Region		
Urban	14	70
Rural	6	30
School		
Primary school	7	35
Junior high school	7	35
High school	6	30
Gender		
Female	5	25
Male	15	75
Education		
Associate diploma holders	1	5
Bachelor’s degree Holders	17	85
Master’s degree Holders	2	10
Years of service as a head teacher		
Less than 3 years	3	15
3–10 years	15	75
11–20 years	1	5
More than 20 years	1	5
Marital status and fertility status		
Married with children	13	65
Married but childless	2	10
Unmarried	5	25

This study employed one-on-one interviews, which were conducted either face-to-face or online. A semi-structured interview outline was formulated which includes three topics as follows:

- What events have triggered your positive or negative emotions in parent–teacher interactions?
- Could you please share whether you attempt to control, regulate, hide these emotions, or express them genuinely? Could you give a detailed account and explain why you did that?
- What impact do you think the management of these emotions in parent–teacher interactions has had on you?

Each interview lasts about an hour and is audio-recorded with the interviewee’s consent. After the interviews, the researchers transcribed each teacher’s interview recordings and sorted them by serial numbers T01–T20 as the original data for the study. This study conducted two rounds of coding. In the first round, 15 interview texts were randomly selected for data analysis. In the second round, the remaining 5 interview texts were used for theoretical saturation.

## 4 Research procedure

### 4.1 Open coding

Open coding refers to the analytical process of identifying concepts in data and discovering their properties and dimensions (Corbin and Strauss, 2008). Firstly, on the basis of abandoning subjective biases and presuppositions, the researchers encoded and labeled the interview texts word by word, coding each word as a node. Then, nodes with less relevance to teachers’ emotional labor in parent–teacher interactions were excluded, and nodes with duplicate meanings were merged to form preliminary concepts. Finally, through continuous comparison and abstraction of these preliminary concepts, 19 initial categories were refined from 81 preliminary concepts. Nvivo12 software was used by the researchers to support this process. Due to space limitations, some open coding examples are shown in Table 2.

**Table 2:** Some examples of open coding.

Initial category	Preliminary concepts	Original sentence
Cultural atmosphere	National culture	I was really angry at first. But then the parents apologized, and I thought, well, let it go. In our Chinese culture, we always try to save face for others, so I could not really stay angry, you know.
	Public opinion culture	Public opinion always unconditionally takes the side of parents, and criticizes teachers from the moral high ground.
	School management culture	School leaders always think it’s the teachers’ fault whenever there’s a conflict with parents, and that we should be criticized. So, even when we are fuming mad, we just have to bite our tongues.
	Teacher culture	I’ve learned a great deal from my colleagues. Just by being around them, I’ve picked up a lot on how to deal with parents.
Educational environment	Educational reform	After the implementation of the ‘Double Reduction’ policy, teachers have to patiently address parents’ misunderstandings and complaints regarding changes in exams, homework, and other aspects.
	Changes in school grade levels	In the lower grades, I have a lot of contact with parents for getting to know them. As the grades go up, the interactions gradually become less frequent. But we are much more familiar with each other. So, I usually express my own emotions more genuinely.
Working condition	Work content	I teach and manage students at work. When I’m off work, I prepare lessons, send out a lot of notices, respond to parents’ phone calls and messages sent in QQ groups or privately. It’s really frustrating when parents do not cooperate.
	Working hours	Sometimes parents call as late as nine or ten at night for stuff that is not pressing, and it really tests my patience.
	Organizational norms	The principal often says to teachers during meetings: “When speaking with parents, you should be careful with your words and tone.”
	Power relations	The status of parents and teachers is not equal. Parents have the upper hand, and teachers nowadays do not dare to offend them.

### 4.2 Axial coding

Axial coding is the process of linking the categories obtained from open coding together (Corbin and Strauss, 2008). Axial coding further induced 19 initial categories obtained from open coding into seven main categories. For example, cultural atmosphere, educational environment and working condition in the initial categories were further induced into emotional labor environment. As shown in Table 3, the seven main categories include emotional labor environment, personal characteristics, emotion competencies, emotional foundation, emotional interactions, emotional labor practices, and emotional labor consequences.

**Table 3:** Main categories formed by the axial coding.

**Main category**	**Initial category**
Emotional labor environment	Cultural atmosphere, Educational environment, Working condition
Personal characteristics	Teacher characteristics, Parent characteristics
Emotional competencies	Emotional intelligence, Communication skills, Ability to enhance emotional intelligence
Emotional foundation	Emotional support, Positional advantage, Common goals
Emotional interactions	Interaction states, Emotional exchange
Emotional labor practices	Emotional management, Emotional expression
Emotional labor consequences	Physical and mental health, Parent–teacher relationships, Working states, Sense of identity

### 4.3 Selective coding and model construction

**Figure 2:**
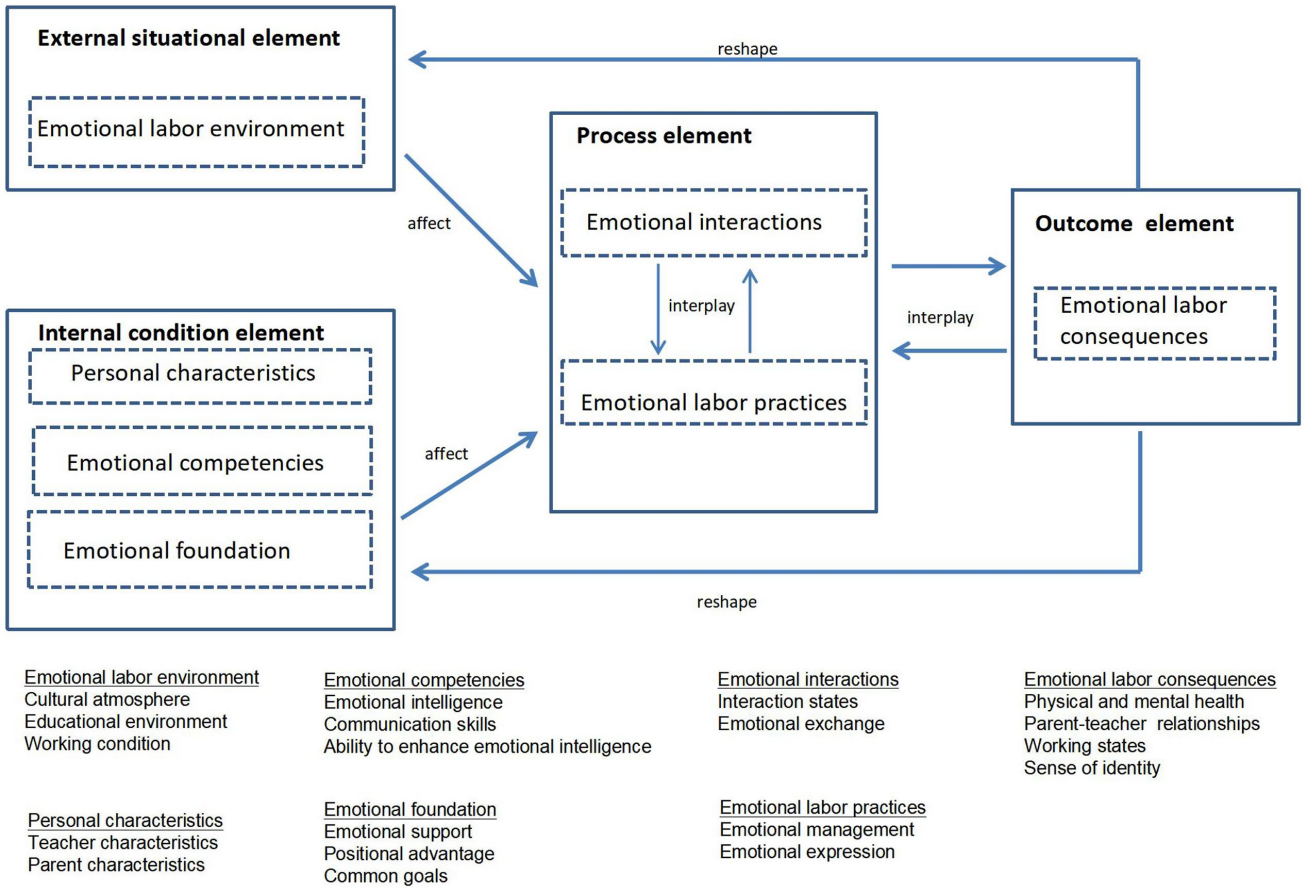
A theoretical model of the key elements of teachers’ emotional labor in parent–teacher interactions.

Selective coding aims to identify the core category which is the central phenomenon around which all the categories are integrated and systematically analyze the relationship between the core category and other categories, so as to develop the theoretical framework (Corbin and Strauss, 2008; Alhassan et al., 2023). By continuously comparing the main categories, this study identified the core category as “the key elements of teachers’ emotional labor in parent–teacher interactions.” After analyzing the interaction mechanisms between these categories, this study has constructed a theoretical model (Figure 2). This theoretical model includes four aspects:

1. Emotional labor environment is an external situational element in teachers’ emotional labor during parent–teacher interactions, which indirectly affects their emotional labor.
2. Personal characteristics are individual factors of teachers’ emotional labor in parent–teacher interactions. Teachers’ emotional competencies serve as the ability condition, while emotional foundation is the relational condition. These constitute the internal condition element that directly affects their emotional labor.
3. Emotional interactions and emotional labor practices mutually influence each other, and together they constitute the process element.
4. The external situational element and the internal condition element jointly influence the process element, resulting in emotional labor consequences, which constitute the outcome element. These results have a reverse impact on subsequent process and to some extent affect or alter the external situation and the internal condition in later stages.

### 4.4 Theoretical saturation test and coding assurance strategies

To ensure the coding reliability and validity, two assurance strategies were adopted. One strategy is a “dividing and then merging” dual-coding approach. Two researchers (one lecturer and one associate professor) independently coded the interview data, then compared, discussed, analyzed, and integrated their coding results to avoid the influence of researcher subjectivity on the coding analysis. Another strategy is theoretical saturation test. This study used the reserved original data to understand theoretical saturation. The 5 reserved interview texts were coded and analyzed according to the previous process. After the categories were formed, they were compared with the formed categories. The results showed that no fresh categories or new relationships were found. This indicates that the theoretical model shown in Figure 2 had reached saturation.

## 5 Explanation of the logic model

### 5.1 Analysis of the external situational element

Emotional labor environment, such as cultural atmosphere, educational environment, and working condition, serves as an external situational element that has an indirect influence on teachers’ emotional labor in parent–teacher interactions. Emotions are the product of social culture. In other words, they are shaped by cultural context. Specifically, national culture, public opinion culture, school management culture, and teacher culture form a multi-layered cultural nested structure, spanning macro, meso, and micro levels. These cultural factors at different levels interact and exert overlapping influences, potentially influencing and constraining teachers’ emotional labor in parent–teacher interactions. For example, Chinese people emphasize face concerns and relational harmony in their emotional expression, especially when interacting with adults (Yin, 2012). Influenced by this national culture, teachers may conceal negative emotions such as anger and use surface acting to “save face” for parents (T12).

Educational environment, such as educational reform and changes in school grade levels, indirectly influences teachers’ emotional labor in parent–teacher interactions. Educational reform affects the emotions of teachers and parents, and especially leads to high levels of stress and anxiety among teachers (Lee et al., 2013; Chen, 2017). Since the implementation of the “Double Reduction” policy in China, parental attitudes towards education have been polarized, with some experiencing heightened anxiety while others finding relief (Qi et al., 2024). This policy mandates that schools reduce students’ academic workload. However, this requirement often clashes with the self-imposed academic pressure from some parents (Lu, 2023). In such context, a lack of mutual understanding between teachers and parents can give rise to a series of negative emotions on teachers, such as helplessness, disappointment, and distress (Chen, 2016; Lee and Yin, 2011). As a result, the burden of teachers’ emotional labor would increase. When students move up in grades, changes occur in parent–teacher interactions. On the one hand, parents tend to show a declining willingness to interact with teachers as their children progress to higher school stages (Wang, 2019); on the other hand, teachers and parents become more familiar with each other as time goes by. These changes indirectly affect teachers’ emotional labor. A teacher in the interview said, “In the early grades, I have to frequently interact with parents to enhance mutual understanding. As the grades go up, the interactions gradually become less frequent, but we are much more familiar with each other. So, I usually express my own emotions more genuinely.” (T15). Her statement shows that changes in school grade levels affect the frequency of teachers’ emotional labor and the emotional labor strategies employed by teachers.

Working condition encompasses aspects such as work content, working hours, organizational norms, and the power relations between teachers and parents (Barutçu and Serinkan, 2013; Hassard et al., 2017). Teachers’ complex and diverse work content, extra working hours, and the norms of parent–teacher interactions in schools indirectly affect teachers’ emotional labor. At present, the use of digital media in home–school interactions blurs the time boundary between teachers’ work and life (Hjorth and Lim, 2012), which may potentially increase their workload and emotional labor burden. Hierarchical power structures between teachers and parents constitute a unique structural work setting (Hargreaves, 2001). The traditional belief that “teachers as experts” tends to place teachers in a high position of authority (Epstein, 2010). By contrast, with educational reforms that aim to commercialize education and empower parents, subtle changes have occurred in the power relations of parent–teacher relationships. Teachers are even regarded as salespeople by parents (Rayner and Espinoza, 2015), and teachers are in a lower position compared to parents. As one interviewee stated, “Parents have the upper hand, and teachers nowadays do not dare to offend them.” (T18). The smaller the power distance, the more genuine the emotion (Zhu et al., 2022). It can be seen that unequal power relations between teachers and parents may influence teachers’ emotional strategies in their interactions.

### 5.2 Analysis of the internal condition element

The internal condition element of teachers’ emotional labor in parent–teacher interactions is composed of three parts: personal characteristics, emotional competencies, and emotional foundation.

#### 5.2.1 Personal characteristics

Teacher characteristics and parent characteristics are individual factors of teachers’ emotional labor in parent–teacher interactions, and they directly affect teachers’ emotional labor. Teachers’ physiological characteristics (such as gender and age), psychological characteristics (such as personality), and social characteristics (such as teaching experience, qualifications) influence their personalized manifestations of emotional labor, including the frequency, intensity, and strategies employed in emotional labor (Antoniou et al., 2006; Basim et al., 2013; Yin et al., 2017; Gonzales, 2022). For instance, female teachers employ surface and deep acting strategies more frequently than male teachers (Akın et al., 2014). Age and experience can also influence teachers’ choice of emotional labor strategies in parent–teacher interactions, as a teacher noted: “As we get older and gain more experience, we can adjust our emotions appropriately. Otherwise, we might get really ticked off by parents sometimes”.

The characteristics of parents as interaction partners can affect teachers’ emotional labor. The characteristics of parents include their educational level, social status, roles in the parents’ committee, expectations for their children, the time they spend communicating with teachers, as well as their willingness and ability to communicate with teachers, etc. (Lasky, 2000; Dotger et al., 2011; Zembylas, 2015). As indicated by the Theory of Interdependence (Rusbult and Van Lange, 2003), teachers’ emotional labor differs based on the various characteristics exhibited by parents. A teacher during the interview mentioned a chairman of the Parents Committee, saying, “Parents always follow the parents’ committee chairman’s lead, so I will be more patient with him.” (T14). It can be seen from this that the leadership role of the parents’ committee chairperson has strengthened teachers’ compliance with emotional display rules.

#### 5.2.2 Emotional competencies

Emotional competence refers to teachers’ emotional intelligence, communication skills, and the ability to enhance emotional intelligence. Although it is usually regarded as an aspect of personal characteristics, the present study identifies it as a main category in parallel with the main category of personal characteristics, so as to highlight the distinction that the former can be proactively developed by teachers while the latter is difficult for them to change. Emotional competencies serve as the ability condition for teachers’ emotional labor in parent–teacher interactions, and directly influence teachers’ emotional labor. High emotional competencies enable teachers to perform well in emotional labor and balance the tension between emotional expression and emotional experience during parent–teacher interactions.

Emotional intelligence manifests as teachers’ ability to understand, manage, and express emotions in parent–teacher interactions (Salovey and Mayer, 1990; Safina et al., 2020; Gonzales, 2022). Emotional intelligence encompasses empathy, cultural sensitivity, reflective ability, expressive ability, and so forth. The higher the emotional intelligence of teachers, the less likely they are to adopt surface acting, and the more likely they are to adopt deep acting and express naturally felt emotions (Yin, 2015). Communication skills refer to teachers’ proficiency in using proactive strategies, maintaining appropriateness, being tactful yet firm, and employing delayed responses, among other tactics, during parent–teacher interactions. Communication skills help teachers gain positive emotional experiences and facilitate the process of emotional labor in parent–teacher interactions. The ability to enhance emotional intelligence is reflected in formal or informal learning, imitation and identification with role models, as well as the communication of expectations and the study of emotion interpretation and expression (Lasky, 2000).

#### 5.2.3 Emotional foundation

Emotional support, positional advantage, and common goals constitute the emotional foundation between parents and teachers, directly affecting teachers’ emotional labor in parent–teacher interactions. Emotional foundation is the prerequisite for establishing and maintaining parent–teacher relationships. It serves as the relational condition for teachers’ emotional labor in parent-teacher interactions.

Emotional support refers to the respect, understanding, trust, and care that parents show towards teachers through verbal or non-verbal means. Since parents are adults who serve as cooperative partners (Gao, 2022), teachers pay more attention to parents’ emotional feedback in parent–teacher interactions. This is different from teacher-student interactions, where the interaction partners are immature educatees. The emotional support from parents will enhance teachers’ emotional energy, sustain their emotional labor input, and improve their emotional labor. The combined positional advantage of being both a head teacher and a parent bridges the emotional distance between parents and teachers. Head teachers have plenty of opportunities to interact with parents. When a head teacher has their own children and is also a parent, it makes emotional understanding between them much easier. The parenting experience that teachers acquire as parents also enhances the trust and appreciation of parents towards teachers (Chang and Hu, 2022). This unique position of being both a parent and a head teacher brings certain advantages, making the psychological distance between teachers and parents closer, facilitating teachers’ emotional labor. Common goals refer to the shared educational objectives of both teachers and parents, which aim to benefit children. These goals are manifested in prioritizing children’s development, showing concern for them, and caring for them. Consistency in parents’ and teachers’ purposes invoked positive emotions (Lasky, 2000), which help teachers foster good parent–teacher relationships, and lay a solid foundation for teachers’ emotional labor. As one interviewee mentioned, “When I’m talking to parents, I just share the emotions I’m feeling. I do not put on a fake act because they get it. They know how much I care about their kids.” (T15).

### 5.3 Analysis of the process element

In parent–teacher interactions, emotional interactions between parents and teachers coexist with teachers’ emotional labor practices, mutually influencing each other, and together they constitute the process element of teachers’ emotional labor in such interactions.

#### 5.3.1 Emotional interactions

Compared with the interactive relationships in commercial service settings, parent-teacher interactions are characterized by greater stability, accompanied by a significantly higher level of emotional involvement on both sides (Yin et al., 2019). In emotional interactions, specific states of parent–teacher interactions and the nature of emotional exchanges shape particular parent–teacher relationships, which directly affect teachers’ emotional labor practices. For instance, such interaction states as interaction duration, interaction frequency, interaction mode, interaction openness, and boundary setting, can affect teachers’ emotional labor in parent–teacher interactions (Farouk, 2012; Chen and Wang, 2011). This is because specific interaction states shape parent–teacher relationships with varying degrees of intimacy, and teachers will perform different emotional labor (such as employing different emotional labor strategies) in parent–teacher interactions based on these different relationships. As a teacher said, “After getting to know some parents better and becoming close with them, I’m more straightforward when expressing my feelings.” (T9).

Emotional interactions are featured by emotional exchanges (Zhai, 2018). Emotional exchange manifests in heart-to-heart interactions, tit-for-tat response, mutual trust, and emotional contagion, all of which influence teachers’ emotional labor practices. Unlike teacher-student interactions, in parent–teacher interactions, both teachers and parents act as highly agentic emotional managers (Cheng and Xin, 2022). For this reason, emotional exchange is more prominent. Positive emotional exchange (such as heart-to-heart interaction) fosters good relationships, provides emotional resources to individuals, and is beneficial to teachers’ emotional labor, while negative emotional exchange (such as tit-for-tat response) impede the development of parent–teacher relationships and exert adverse effects on teachers’ emotional labor practices. For example, a teacher in an interview said, “When parents avoid the question and complain to me, I just complain back to them, and it just fizzles out in the end.” (T10). Based on the distinct states of interactions and the unique nature of emotional exchange between teachers and parents, different parent–teacher relationships are constructed and distinguished (Cheng and Xin, 2022). These relationships, in turn, shape teachers’ differential emotional labor practices. In other words, the relational dimension offers a lens through which teachers’ emotional labor in parent–teacher interactions can be understood.

#### 5.3.2 Emotional labor practices

Emotional management and emotional expression constitute the core aspects of emotional labor practices. In parent–teacher interactions, teachers’ emotional management includes changing cognition, controlling attitude, managing facial expressions, and adjusting tone, among others. Teachers’ emotional expression includes expression of naturally felt emotions, disguised expression, and refusals to express. As mentioned above, expression of naturally felt emotions does not make any emotional modification (Yin, 2012). Disguised expression can be categorized into surface acting and deep acting. In both cases, individuals display emotions that do not truly reflect their inner emotions (Yin, 2012). The former refers to the strategy in which teachers fake unfelt emotions or hide felt emotions in order to display the appropriate emotions required by their job (Yin, 2012). The latter means the process in which teachers try to modify their felt emotions by employing certain cognitive techniques (e.g., distraction and self-persuasion) to achieve the desired emotional displays (Yin, 2012). When asked how teachers manage emotions in parent–teacher interactions, interviewees shared their strategies. One interviewee said that she would pretend to be angry to draw parents’ attention (T6), which is a case of surface acting. Another interviewee said that he would sometimes change his perception to regulate his emotions, in other words, adopt deep acting. He said, “I was a bit annoyed at first, but then I realized that parents have their own struggles and they did not mean to cause trouble, and so I calmed down” (T8). When teachers refuse to express their emotions to parents, this is considered a refusal to express. As a teacher said, “Some parents are really hard to get along with, so I just keep my distance from them.” (T11). The findings reveal that refusing to express emotions is also a strategy of teachers’ emotional labor in parent–teacher interactions, which expands previous understandings that such strategies are limited to surface acting, deep acting, and natural expression. This phenomenon precisely stems from the characteristics of parent–teacher interactions. Unlike teaching-and-learning relationship between teachers and students, the relationship between teachers and parents is essentially a cooperative one (Gao, 2022). Although such cooperation is a requirement of schools, when it comes to a specific parent, teachers can choose to reduce communication with or even refrain from communicating with that particular parent for some reasons.

Teachers’ emotional labor practices are not only influenced by parent–teacher emotional interactions but also have a reverse effect on it. Emotional labor takes social interactions as its medium, and the product of emotional labor is an intangible interactive experience. Teachers’ positive emotional labor practices can provide both teachers and parents with a favorable interactive experience, evoke their confidence and enthusiasm for emotional interactions, and thereby promote emotional interactions between the two parties. Teachers’ negative emotional labor practices, on the other hand, are contrary to this. For instance, a teacher reported that positive emotional management enabled her to resolve a conflict between parents and teachers triggered by school uniform quality issues, and that the emotional interactions were further enhanced through emotional contagion between them (T2).

### 5.4 Analysis of the outcome element

Teachers’ emotional labor process in parent–teacher interactions affects teachers’ physical and mental health, parent–teacher relationships, teachers’ working states and their sense of identity, resulting in positive or negative emotional labor consequences, which are the outcome element of teachers’ emotional labor in parent–teacher interactions. The outcomes have a reverse impact on subsequent process and to some extent affect or alter the external situation and the internal condition in the later stages.

When teachers’ emotional labor results in the prolonged depletion of psychological resources without timely replenishment, it can precipitate a range of symptoms, including anxiety, depression, stress, and occupational burnout (Martínez-Monteagudo et al., 2019), which has harmful effects on teachers’ physical and mental health. Regarding the impact of emotional labor on teachers, a female interviewee believed that the long-suppressed resentment had caused her to develop breast nodules (T16). Emotional labor also affect parent–teacher relationships, teachers’ working states and their sense of identity. Positive emotional labor create opportunities for workers to establish mutual trust and respect with others, fostering meaningful emotional connections. This helps to harmonize parent–teacher relationships, boost teachers’ work enthusiasm, enhance teachers’ work performance, and enable teachers to continuously affirm themselves and cultivate a cohesive sense of identity (Liu, 2019). This verifies Hargreaves’ assertion that emotional labor has not only exchange value but also use value (Hargreaves, 1998). Excessive and negative emotional labor may harm parent–teacher relationships, disrupt teachers’ working states, and compromise their sense of identity. For example, a teacher said, “I do not want to pander to parents against my will, but I’ve got no choice. And it’s making it really hard to focus on my work (T7).” And the teacher even entertained the idea that “I am not suitable to be a head teacher (T7).” Notably, emotional labor strategies play a certain moderating role in determining the nature of emotional labor outcomes. It is believed that deep acting, compared to surface acting, has a more positive impact on workers’ physical and mental health and their subsequent work performance when they are in a negative emotional state (Ma and Huang, 2006). In the context of parent–teacher interactions, different emotional labor strategies employed by teachers can thus have either positive or negative effects on their health, parent–teacher relationships, and sense of identity.

The consequences of teachers’ emotional labor are triggered by emotional labor practices, which in turn influence subsequent teachers’ emotional labor practices. Positive consequences of emotional labor encourage teachers to invest in emotional labor, affirm their choice of strategies, and bring positive impacts to subsequent emotional labor practices. The negative consequences of emotional labor may encourage teachers to reconsider and modify their emotional labor practices, or they may cause teachers to conduct emotional labor in a perfunctory manner or even avoid emotional labor. A teacher said, “The relationship with parents is hard to put into words. Sometimes, it’s better to keep a distance, just mind my own business and let it be” (T7).

To some extent, the consequences of emotional labor also reshape the emotional labor environment, personal characteristics, emotional competencies, and emotional foundations (Schutz and Lee, 2014; Pekrun and Schutz, 2007). On the one hand, social structure is the result of collective action. The external emotional labor environment is not independent of teachers’ emotional labor, and its consequences subtly shape the emotional labor environment (Pekrun and Schutz, 2007). On the other hand, personal characteristics, emotional competencies, and emotional foundation are inseparable from the accumulation of emotional labor in the past. The consequences of teachers’ emotional labor in parent–teacher interactions will always permeate the formation of the internal condition. Based on this, the external situational element, the internal condition element, the process element, and the outcome element of teachers’ emotional labor in parent–teacher interactions constitute an interrelated and dynamic organic system.

## 6 Discussion and conclusion

This study explores the key elements and theoretical logic of teachers’ emotional labor in parent–teacher interactions based on grounded theory, and finds that: emotional labor environment, personal characteristics, emotion competencies, emotional foundation, emotional interactions, emotional labor practices, and emotional labor consequences are the key elements of emotional labor. Emotional labor environment serves as an external situational element that has an indirect influence on teachers’ emotional labor in parent–teacher interactions. Personal characteristics, teachers’ emotional competencies and emotional foundation together constitute the internal condition element that directly affects teachers’ emotional labor in these interactions. Emotional interactions and teachers’ emotional labor practices, which serve as a process element, generate positive or negative emotional labor consequences. These outcomes not only have a reverse impact on subsequent processes but also, to some extent, affect and even alter the external situation and the internal condition in later stages. Based on these findings, a theoretical model is constructed.

This research has some theoretical implications, as it expands the study of key elements of teachers’ emotional labor in parent–teacher interactions. The identification of elements such as emotional labor environment, personal characteristics, emotion competencies, and emotional labor consequences validates Grandey’s conceptual framework (Grandey, 2000). Furthermore, this study places special emphasis on the uniqueness of teachers’ emotional labor in parent–teacher interactions. Some categories (including emotional support, positional advantage, common goals, interaction states, emotional exchange) overlooked by scholars are identified, and elements of emotional foundation and emotional interactions were further extracted. This study on teachers’ emotional labor in parent–teacher interactions breaks through the limitation of merely focusing on organizational and individual levels (Brotheridge and Grandey, 2002). It centers on the interpersonal level, revealing that emotional foundations, as a relational condition, and emotional interactions, as a relationship-building process, play significant roles in this context. This highlights their importance and enriches relevant research from the relational dimension (Zhang, 2022). This study not only fill the research gap in teachers’ emotional labor within parent–teacher interactions (Lasky, 2000; Kang et al., 2023) by summarizing its key elements but also, through the construction of a theoretical framework, identifies the dynamic circular relationships among these key elements, systematically reveals the process mechanism of such emotional labor, and thereby provides an analytical framework that can be referenced for future related studies.

In addition to the theoretical values mentioned above, these findings can provide some insights for school administrators, teacher educators, and teachers.

Firstly, school administrators should recognize the significance of teachers’ emotional labor in home–school cooperation and create a supportive emotional labor environment for them. Specifically, it is advocated to foster a school management culture and teacher culture that protect teachers while enhancing their power (Pollard, 1982). School administrators should also formulate policies to reduce the pressure on teachers from educational reforms, ensure a balanced workload, and take into account the emotional labor involved in teachers’ interactions with parents. Schools should regularly organize activities for parents and teachers to promote positive interactions. They should also pay attention to the consequences of teachers’ emotional labor, such as impacts on their physical and mental health, and actively seek feedback from teachers regarding their emotional labor experiences. Building on this feedback, schools administrators should promptly reflect on their management practices and implement targeted interventions to lighten teachers’ emotional labor burden and facilitate parent–teacher cooperation.

Secondly, teacher educators should attach importance to cultivating teachers’ emotional competencies. These competencies are key qualities that make teachers less likely to be replaced by artificial intelligence in the age of intelligence. They form the core of teachers’ professional development and permeate the entirety of their careers. In both pre-service and in-service teacher training, teacher educators should design specialized courses to enhance such competencies (Bahia et al., 2013; Yin, 2016). These courses should be tailored to teachers’ personal characteristics, such as gender, age, personality, and teaching experience, and use differentiated approaches to better prepare them for communicating with parents from diverse backgrounds.

Finally, teachers should optimize their emotional labor in parent–teacher interactions from a relational dimension. Teachers should strengthen the emotional foundation by cherishing parents’ emotional support, appropriately utilizing teachers’ positional advantages, and reinforcing the common goals between teachers and parents, so as to build a healthy and harmonious parent–teacher relationship for home–school cooperation. In addition, teachers should maintain appropriate emotional interactions with parents, address their emotional needs, and create opportunities for positive emotional exchange. Of course, all of these require teachers to possess good emotional competencies. To this end, teachers should enhance their emotional competencies through such approaches as systematic learning, consulting with peers, and reflecting on practice (Lasky, 2000; Cil and Dotger, 2017). These practices will facilitate teachers’ emotional labor in parent–teacher interactions.

## 7 Limitations and future research

This study has some limitations that future research could address. First, the study participants are primary and secondary school teachers who are head teachers in China, and thus do not represent teachers in other cultural contexts. The specificity of Chinese culture may limit the applicability of the research results across cultures. It is recommended for the future research to include teacher groups from other countries, and explore the impact of their unique cultural backgrounds on teachers’ emotional labor in parent–teacher interactions. Another limitation is that this study adopted a qualitative grounded theory approach, and therefore has limited generalizability. Large-scale quantitative studies should be conducted to validate the findings of this study.

## Data Availability

The raw data supporting the conclusions of this article will be made available by the authors, without undue reservation.
